# How can a 6-week training course shape mental healthcare professionals’ understanding of mindfulness? Experiences at Weskoppies Psychiatric Hospital

**DOI:** 10.4102/sajpsychiatry.v27i0.1489

**Published:** 2021-03-23

**Authors:** Nathalie H. Negus, Gerhard Grobler

**Affiliations:** 1 Department of Psychiatry, Faculty of Health Sciences, University of Pretoria, Pretoria, South Africa

**Keywords:** healthcare workers, mindfulness, skills, training, Weskoppies Psychiatric Hospital

## Abstract

**Background:**

Mindfulness-based practice has gained increasing attention in the mental health community over the last four decades, and many studies have explored the evidence of its various benefits among healthcare users and providers alike. However, there remains limited research regarding the understanding of mindfulness among mental healthcare professionals. This poses the question: how much do mental healthcare professionals really know about mindfulness, and can self-practice increase the understanding of these providers?

**Aim:**

This descriptive or exploratory case study aimed to explore the understanding of mindfulness amongst 15 mental healthcare professionals.

**Setting:**

The study took place at Weskoppies Psychiatric Hospital.

**Method:**

The study was conducted following a 6-week training course in which the participants were taught, and carried out mindfulness-based practices and techniques. The study also explored the following: (1) the healthcare workers’ experiences, benefits and challenges regarding the consistent practice of mindfulness and (2) their confidence when explaining the concept of mindfulness, and the practices learned, to other colleagues and patients. Data were collected in the form of semi-structured interviews with the participants, 4–6 weeks after completion of the training course.

**Results:**

Three main themes were identified: (1) understanding of mindfulness expanded with practice; (2) unexpected experiences during the mindfulness course; and (3) experience caused partial gains in confidence and skills. Overall, 15 subthemes were derived from the data collected.

**Conclusion:**

Self-practice of mindfulness can increase one’s understanding of the concept and the confidence to teach informal techniques. More research is needed to determine how the design and duration of such training could impact this understanding and confidence.

## Introduction

Mindfulness is a practice that has been a part of the Buddhist faith for centuries, and it has become a popular topic of research amongst Western mental healthcare within the last 30–40 years. Professor Jon Kabat-Zinn, who developed a programme of mindfulness-based stress reduction (MBSR) in 1979, described mindfulness as ‘purposefully paying attention to the present moment, in a curious, open way, without judgement’.^1^ The effects of mindfulness-based practices upon users have been explored over the last 40 years in the context of various illnesses and conditions and have not only shown promising benefits for people with mental healthcare disorders but also for people with a number of physical ailments and conditions, such as fibromyalgia, sleep disturbances and chronic pain.^2,3^ Amongst the various benefits and outcomes that have been described in studies on mindfulness practice, positive impacts have been noted upon participants’ empathy, self-compassion, esteem and self-regard, emotional regulation, attention, stress perception, internal and external awareness, and coping skills within pressured environments.^2,4,5^ Prosocial behaviours were also noted to improve with various forms of mindfulness-based practice, such as improved awareness of and attentiveness to others’ needs and feelings. Listening skills, increased sensitivity to non-verbal cues and a greater sense of cohesion, or ‘shared humanity’, are further outcomes noted with the practice of mindfulness in various forms.^6,7^ It has been further postulated by various neurobiology studies that the self-awareness, self-regulation and self-transcendence that are developed through mindfulness meditation are associated with the modulation of certain frontoparietal networks associated with self-perception and narration.^8^

In recent years, the applications of mindfulness-based practices have extended to those who provide healthcare as well as those receiving it. In a 2014 systematic review conducted by P. Morgan et al., focused on the literature describing healthcare professionals’ experiences of mindfulness training, reports reflected shifts in mindset amongst the involved healthcare professionals. An example of such changes included not only caring for others, but also caring for oneself, as well as an increase in self-compassion.^9^ An increased level of relatability and compassion for their patients’ conditions was also reported.

Despite the relatively significant amount of literature exploring the benefits and outcomes of mindfulness, and its related programmes, there has been, until recently, little insight into *what* mindfulness is in the eyes of its participants: *how* do they understand it as a concept?

To complicate matters further, there has been added scrutiny by some researchers into the potential *harmful* effects of mindfulness meditation. Not only have some studies shown that mindfulness meditation had no greater merit than other forms of therapy for patients with depression, but research has explored the possibility that, in tapping into previous negative and possibly traumatic experiences, without the right support in place, a patient can experience an *increase* in stress, anxiety and even depression.^10^

The concept of mindfulness, though a newer and seemingly promising intervention in the field of mental health, is still relatively undiscovered by many healthcare professionals, and in order to develop and teach the valuable skills that this practice can offer, healthcare professionals need to first understand and experience it.

Both quantitative and qualitative methodologies have been used in mindfulness research. Various self-reporting scales have been constructed to attempt to measure mindfulness skill or awareness. However, doubts and concerns have been raised regarding such tools of measurement. Examples of such concerns include the lack of external resources by which to determine the validity of these kinds of scales, the lack of convergent validity between different scales, and the wide discrepancy noted between such reports of mindfulness competency versus the actual behaviours practised.^11^ Qualitative research, by its nature, attempts to gain an understanding of concepts such as mindfulness from an *experiential* point of view, across a wide range of ages and across various different occupations. This is of particular interest amongst healthcare professionals, where care and compassion is encouraged in their interactions with patients but often neglected in their own spheres.

There are various examples of studies in current literature that attempt to understand the experiences of healthcare professionals who are trained in mindfulness. A qualitative study conducted in the Netherlands interviewed medical residents who participated in a MBSR course at Raboud Medical University. On qualitative exploration of the influence the course had on these residents, themes of increased self-awareness, self-reflection, acceptance and resilience emerged. However, this study did not specifically explore the influence that MBSR training had on the residents’ understanding of mindfulness as a practice nor their ability to share those practices with others.^12^

The same can be said of a 2014 study conducted by Lyddy et al., exploring the experiences of healthcare professionals who took part in a training programme derived from the Tergar Meditation Community’s ‘Joy of Living’ programme. Data emerged that showed the variability of the healthcare professionals’ application of the training, depending upon other priorities, time and space challenges, and identity factors but not upon the development of their understanding of *what* mindfulness *is*.^13^

Whilst the number of studies related to the experience of healthcare professionals in mindfulness training is growing, limited, if any, research has been conducted amongst professionals working in mental health services, and even fewer studies have been done on this concept in a South African setting, where high patient numbers and fewer resources often add to the stress and pressure experienced by staff members. It would be of great interest to review the degree of understanding that can be cultivated, or expanded on, amongst healthcare workers in this environment.

Weskoppies Psychiatric Hospital (WKH), a specialised mental healthcare facility situated in Pretoria, South Africa, is such a setting in which mindfulness can have significant influence upon both mental healthcare users and practitioners alike. A 6-week course, offering training in mindfulness-based practices to staff members working at this facility, was held in the months of April and May 2017 by two senior clinicians (psychiatrists) with experience in mindfulness-based practice.

The aim of this study thus was to explore participants’ understanding of mindfulness following the training course, as well as their degree of confidence in sharing the skills they learned with other colleagues and patients under their care. Furthermore, this study also explores the experiences of each participant regarding the practice of mindfulness itself: benefits gained, challenges faced and changes noted regarding one’s perspective.

## Methodology

### Study design

The study design was a qualitative exploratory and descriptive case study, a research method whereby specifically targeted contemporary phenomena are discussed and investigated in the context of real-life examples.^14^ Using this method of research, multiple sources can be utilised for data collection, from interviews and observation of cases to journal entries, questionnaires and historical documents.^15^ In the case of this research study, the context comprised the multiple viewpoints of mental healthcare workers following their participation in the 6-week mindfulness training course provided at WKH.

### Setting

The study took place at WKH, in Pretoria, Gauteng, South Africa, as did the preceding training course.

### Sample selection and consent

The training course was advertised at WKH over a period of 8 weeks and took place over 6 weeks. Sessions were held twice a week, providing a total of 12 sessions of training in mindfulness-based practices. The training course content and structure were derived from Jon Kabat-Zinn’s MBSR course and included an introductory session explaining mindfulness. The following 11 sessions comprised the guided practice of various exercises, such as body scanning, breath meditation, sound meditation and thoughts and emotions-related meditation. Homework was given to participants in between sessions, in the form of practising exercises learned in sessions, using mindfulness in day-to-day activities and compact discs of guided meditation to assist them. Feedback was discussed during sessions and experiences shared between the participants. Each session was an hour in duration and included discussion of experiences amongst the group after each exercise. Both the psychiatrists conducting the course had experienced mindfulness through self-study and self-practice, using resources from various mindfulness experts such as Jon Kabat-Zinn, Jack Cornfield and Bruno Cayoun. Although they agreed to assist in informing the course attendees of this study, they did not supervise the implementation of this study, thus avoiding conflict of interests.

Staff members who participated in the training course were invited to participate in the research study. All staff members attending the training course were provided with an information leaflet at the start of the first session, explaining the outline and reasons for the study, the requirements of each participant, and the steps to ensure confidentiality of their personal demographics and information given for the study. Those who provided written informed consent participated in the research study on completion of the training course. The inclusion criteria for participation were:

- employed as a healthcare professional at WKH
- able to give informed consent
- completed at least 50% of the sessions (i.e. six sessions) of the mindfulness training course.

### Data collection and analysis

The data required for this study, namely, the experiences and understanding of the healthcare workers who met the above criteria following the training course, were collected via semi-structured interviews, 4–6 weeks after the course had ended. One interview was held with each individual participant, with the assistance of predetermined questions, which the participant could answer with as much or as little elaboration as they chose, with the possibility of deviation within the theme of their experiences and understanding of mindfulness. The questions compiled to assist these interviews were drawn from other established inventory scales (such as the Freiburg Mindfulness Inventory scale) related to the effects of mindfulness; however, they were not wholly based upon them. Because of the specific aims of the study, especially the aim of exploring participants’ *understanding* of mindfulness, and not only their experiences, the questionnaire was tailored to the needs of the study objectives and discussed amongst the authors and course conductors. The interviews were held by Dr N.H. Negus and recorded electronically. The recordings were later transcribed verbatim by a professional transcription service for further analysis. The data, in the form of the transcribed interviews, were analysed by way of thematic analysis, by the primary researcher of the study. After thorough familiarisation with the content of the interviews, important information was highlighted and common themes and descriptions were then grouped together under the same highlighted colour. These groups were then analysed individually and repetitively until themes and subthemes could be extracted and condensed from the data. These formed the overall ‘answers’ to the objectives and aims of the study.

### Ethical considerations

Ethical approval to conduct the study was obtained from the Ethics Committee of the University of Pretoria (UP) on 29 June 2017 (protocol no. 239/2017), prior to the commencement of data collection and no ethical concerns were noted throughout the course of the research process.

## Results

The mindfulness training course completed by various staff members at WKH began the course with 16 participants from various healthcare roles. All 16 participants agreed to take part in this study; however, only 15 of the participants met the criteria, with one of the healthcare workers only completing two sessions of the 6-week course. Of the 15 participants, one was a psychiatrist, two were psychiatrists in training (‘registrars’), 10 were nurses and two were social workers. The number of sessions completed by each participant ranged from nine to 12 sessions. The duration of the interviews held with each participant was on average 30–60 min each, and their transcribed documents ranged between 1905 and 6785 words each. From the extensive data that were captured from the 15 interviews, three main themes, with three to five subthemes each, were derived.

### Understanding of mindfulness expanded with practice

This theme was developed around the exposure of the participants to mindfulness, or lack thereof, prior to the training course held and illustrated how individual understanding of mindfulness was shaped by the participants as knowledge was gained upon the practice.

#### Little to no prior exposure to mindfulness

It was noted by the majority of participants that mindfulness was a topic they had scarcely heard of, and for many of those who had heard of the concept in literature, a definition or description of the concept was never provided. Approximately one-third of the participants reported some prior exposure to the concept of mindfulness, either through hearing or reading about it, but only one of these participants had previously taken a basic course and practised mindfulness techniques. Of those who had briefly been acquainted with the concept, most of their information was self-taught, through reading materials, other overlapping self-development topics (such as resilience) or spirituality.

#### Pre-notion of awareness, present focus and self-control

Despite the scanty exposure of many of the participants to mindfulness in the healthcare system, many of them could offer a fairly accurate description of some of its principles. Many of the participants felt that mindfulness involved awareness, but only directed towards internal *or* external factors. A number of the participants emphasised the ability to focus upon the present moment and that mindfulness strengthens the ability to have more control over one’s attention and emotions.

#### Awareness needed in everything

Over the course of the training programme, however, the understanding of these participants seemed to expand, to incorporate in greater fullness one of the essences of mindfulness: the awareness of *everything* within the present moment, both within oneself and surrounding oneself.

#### Realisation of non-judgemental approach

A number of the participants learned that a core concept of mindfulness involves not only becoming aware of oneself in terms of thought, emotion and action, but choosing not to judge those elements and instead simply accepting them for what they are in the moment. Participant 6 described the practices as follows:

‘[*M*]indfulness training … makes you more aware of who you are, of what you’re feeling in that moment … and then to acknowledge how you’re feeling and not to feel bad at that … it’s OK to feel negative emotions’. (Participant 6, female, social worker)

#### Lifestyle, not only techniques

An interesting development in the experience and understanding of mindfulness by participants was in realising its value as a mindset and perspective, rather than simply a group of skills. As described by participant 8,

‘If you just read in books, then you don’t feel it so much in your whole being … that mindfulness is, is more about your being … not so much about your doing, but really the being’. (Participant 8, female, social worker)

### Unexpected experiences during the mindfulness course

Although the majority of experiences of the course were viewed as positive, many participants voiced that their experiences differed from their expectations in certain ways. A number of the participants initially expressed the recurring desire to build upon their knowledge of mindfulness and how to practise it. Some also hoped that learning how to practise mindfulness would be a tool to assist them in controlling their own emotions, coping with stressful situations or improving their concentration. These expressed desires were categorised under the subtheme *Expectancy for knowledge, self-control and coping skills.* Throughout the training sessions, participants not only noted changes in these areas of interest but also reported on other experiences regarding the course and training that they did not anticipate.

#### Increased awareness and understanding of self, others and environment

Most of the participants experienced an expansion of their awareness, either of their own emotional and mental state or of the external world around them. One healthcare worker, participant 5, described her own experience of increased awareness:

‘[*A*]ll of a sudden I wonder where am I now; it’s a road that I am travelling 20 years now … now you could hear the birds and sounds and an aeroplane flying and all those kind of things… it never bothered me in the past or I was never aware of it’. (Participant 5, female, nursing sister)

Another participant shared that a new experience for her was that of becoming aware of her body’s reactions to stressful situations:

‘[*I*]t was a discovery that you realised that before you respond to a situation you can actually take time to pause … I become aware of … how my body is physically biologically responding’. (Participant 4, female, mental healthcare nurse)

#### Understanding the need for self-care and kindness

Along with the increased internal or external awareness that was noted by many of the healthcare workers, there was a second revelation that seemed even more prevalent amongst them: an unexpected need for self-care. Some participants realised that although they may be used to taking care of others, treating themselves with care was less of a priority. Fourteen out of the 15 participants found themselves struggling to stay awake during some of the meditative sessions, bringing to many the realisation of just how rarely one takes the time to stop and reflect in day-to-day life. These experiences also included some becoming aware of their boundaries and inner dialogue.

#### Improved self-control, communication, objectivity

A number of participants noted, as the mindfulness practice progressed, that they experienced less irritability with previously stressful interactions and found themselves able to empathise with other parties and understand others’ perspectives. This enabled some of the participants to communicate more effectively and to ‘respond, rather than react’.

#### Challenges with lack of structure, time, consistency and unpleasant emotions

Despite the positive outcomes throughout the course, there were challenges experienced by many participants. The course itself, for example, was based largely upon the practice of mindfulness-based exercises. Some, such as one called the ‘body scan’, were difficult for the participants to grasp, either because of the abstract nature of the activity or the difficulty in maintaining focus for an extended period of time.

Furthermore, despite acknowledging their positive effects, some participants found it difficult to persist with these exercises on a habitual basis, because of the time that it took to carry out the exercises, or the pace of daily life.

Thirdly, for some, the awareness of *negative* emotions (such as anger or sadness) was difficult to tolerate during the practice of mindfulness.

### Experience caused partial gains in confidence and skills

Apart from assessing the experiences and evolving understanding of the participants regarding mindfulness, our final objective was to assess the degree of confidence with which they felt able to share the skills and concepts that they had learned with other colleagues and clients. This final point of focus shed light on the above theme, summarised under the following three subthemes.

#### Basic concepts only

A more in-depth understanding of mindfulness was clearly obtained by many of those who attended the training course. The sharing and explaining of this concept to others, therefore, seemed to be a task that the majority of participants could do with confidence, even though this confidence did not seem to extend to the specific techniques that they themselves had practised.

#### Informal rather than formal practice

It seemed that the majority of the participants found informal applications of mindfulness practice more useful and realistic to implement than the formal techniques. Examples given of this include focusing on one’s breathing and pausing to become aware of external and internal factors.

#### Difficulty with low-functioning users

This particular subtheme was a topic not necessarily explored with every participant; however, it lent an interesting perspective on how applicable they felt mindfulness would be across the general population. Though mindfulness as a whole was generally agreed to be beneficial to everyone, there seemed doubt that it could be as beneficial to those with certain impairments or illnesses, such as patients with psychosis, or those who ‘don’t have a high emotional intelligence level’ (Participant 14).

## Discussion

There exists, currently, very little research on the effects of *teaching* mindfulness to participants – not only on how the application of its practices can shape one’s understanding of the theory behind them, but on one’s ability to explain this theory to others. A 2013 systematic review attempted to identify the existing tertiary facilities that sought to implement some kind of mindfulness teaching into medical and dental student programmes^16^ and found 14 facilities in the search, mostly in the United States of America and Canada. However, this article posed questions regarding the very same lack of research emphasised above: how and when is it effective to teach mindfulness to medical professionals, and how is that understanding best maintained? Similar questions have been posed regarding the use of mindfulness training in clinical psychology programmes.^17^ A study focused upon the use of mindfulness practice by potential teachers of mindfulness-based interventions emphasised the importance of such teachers to ‘embody the practice’ of mindfulness and develop ‘pedagogical competencies’.^18^ However, with the training still underway at the time that study was published, little is mentioned about the resulting development of such competencies. This study has attempted to focus upon multiple facets of the mindfulness training experience – the challenges, changes and perspectives of mental healthcare workers practising mindfulness – but also to uniquely explore the potential ways in which such training may empower them to transfer this knowledge to others. The importance of a holistic understanding of mindfulness, in order to practise or teach it, is repeatedly noted in the literature – lest, as explained effectively by psychologist Khong,^19^ ‘mindfulness become simply another technique or intellectual theory, instead of understood as a way of *being*’. This study provides a further specific perspective in analysing the developing understanding of professionals working in a specialised mental healthcare field – a perspective that seems surprisingly scanty in the current literature. Certain themes extracted from the experiences of the study’s participants are similar to the findings of other qualitative studies that explore experiences of mindfulness-based practice. Examples of this can be found in a 2015 study that focused primarily upon the shifting intentions of participants in practising mindfulness.^20^ Subthemes arose related to a cultivation of compassion, a realisation of changes in behaviour and awareness, and a redefining of their definition of mindfulness meditation. These correlate strongly with themes discussed in this study, such as *Understanding the need for self-care and kindness* and *Increased awareness and understanding of self, others and environment.* Another theme of interest that was reflected in a 2014 qualitative review of healthcare professionals involved in a MBSR course was the realisation that a degree of frustration or distress forms an integral part of learning mindfulness practice.^21^ Similar challenges were expressed by a number of participants in the study of this article, in the form of unpleasant emotions and difficulties in knowing *how* to carry out such practices.

With regard to the effects of the 6-week training course upon participants’ understanding of mindfulness, and their ability to share its concepts, it is suggested that the course gave its participants a richer, more expanded understanding of what mindfulness is. Of particular interest was the realisation of the concept of ‘non-judgement’, or acceptance: a key pillar in the Buddhist psychology that underpins the concept of mindfulness.^20^ Furthermore, there was a developing understanding amongst participants of mindfulness as a state of *being* more than doing, as part of a lifestyle rather than techniques alone. This understanding in itself may have more of a positive impact upon one’s ability to explain mindfulness to others, beyond one’s ability to explain formal practices. It was noted that many of the participants felt much more comfortable sharing examples of informal application of mindfulness, through daily habits such as driving, eating or focusing upon one’s thoughts or emotions. Such ‘informal’ applications have greater capacity for adaptation to suit various population groups. An example of this was reflected in another 2014 study, in which patients with mild-to-moderate intellectual disability were involved in a mindfulness course with a focus mainly upon sense-related exercises (fruits, scented candles, music, etc.).^22^ The outcomes of the study showed that these patients had the capacity to develop an understanding of mindfulness and experience its related benefits – an interesting comparison to the theme noted amongst our participants that ‘low-functioning users’ may experience more difficulty in benefitting from mindfulness practice.

There were some limitations in this study. Firstly, most participants were not English-first language speakers, and all interviews were conducted in the English language. Although all participants were fluent in English, this could have inhibited or limited their ability to fully express their experiences of the course. Secondly, though the analysis of the data was reviewed by a second supervisor, the thematic analysis itself was carried out by a sole researcher. The accuracy and validity of the themes uncovered may have been more strongly validated by the analysis of more than one researcher, as well as through the use of qualitative analysis software (no such software was used during analysis of the data).

## Conclusion

The concept of mindfulness, and mindfulness-based practice, holds many benefits for various members of the medical and mental healthcare community. It may be beneficial for medical professionals (and specifically, mental healthcare professionals) to understand the basic foundations of mindfulness and develop some competencies in its application to daily living. Though much research exists upon the effects of mindfulness practice amongst healthcare professionals, there remains little evidence as to the effects of mindfulness practice upon healthcare workers’ abilities to explain and teach mindfulness to others. This study reveals that training in self-practice could lead to an expansion of basic understanding of mindfulness and an increase in confidence to teach informal techniques of mindfulness. More qualitative research is needed into the ways that various designs and durations of mindfulness training can impact the teaching competency and depth of understanding of this concept amongst healthcare professionals.
